# Influence of Perceptual Saliency Hierarchy on Learning of Language Structures: An Artificial Language Learning Experiment

**DOI:** 10.3389/fpsyg.2016.01952

**Published:** 2016-12-21

**Authors:** Tao Gong, Yau W. Lam, Lan Shuai

**Affiliations:** ^1^Haskins Laboratories, New HavenCT, USA; ^2^Center for Linguistics and Applied Linguistics, Guangdong University of Foreign StudiesGuangzhou, China; ^3^Department of Linguistics, University of Hong KongHong Kong, China

**Keywords:** perceptual saliency hierarchy, artificial language learning, syntax, learning bias, diversity

## Abstract

Psychological experiments have revealed that in normal visual perception of humans, color cues are more salient than shape cues, which are more salient than textural patterns. We carried out an artificial language learning experiment to study whether such perceptual saliency hierarchy (color > shape > texture) influences the learning of orders regulating adjectives of involved visual features in a manner either congruent (expressing a salient feature in a salient part of the form) or incongruent (expressing a salient feature in a less salient part of the form) with that hierarchy. Results showed that within a few rounds of learning participants could learn the compositional segments encoding the visual features and the order between them, generalize the learned knowledge to unseen instances with the same or different orders, and show learning biases for orders that are congruent with the perceptual saliency hierarchy. Although the learning performances for both the biased and unbiased orders became similar given more learning trials, our study confirms that this type of individual perceptual constraint could contribute to the structural configuration of language, and points out that such constraint, as well as other factors, could collectively affect the structural diversity in languages.

## Introduction

Physical objects can be discriminated by visual features such as color, shape, and texture. Human eyes are essentially light receptors, and thus, color or brightness information requires little cognitive load for processing, thus becoming the strongest cue for visual perception. In terms of evolution, the alimentary “niche” also enhanced color perception in humans and other primates (Dominy and Lucas, 2001; Melin et al., 2012). Difference in color or brightness enables humans to perceive additional features such as shape and textural pattern. Per these fundamental features (color, shape, and texture), psychological experiments have explicitly shown that: random variations in color interfere with viewer’s ability to identify shapes, but variations in shape have no explicit effects (in terms of judgement accuracies and reaction times) on color discrimination (Callaghan, 1990; Healey, 2000); and random variations in color or shape interfere with viewer’s identification of visual patterns of texture, but not vice-versa (Treisman, 1985; Healey and Enns, 1999). This evidence reveals a *perceptual saliency hierarchy* (PSH, the relative conspicuousness of various visual features at first exposure, Healey and Enns, 2012), which states that in normal visual perception of humans, color information appears to be more salient than shape information, and shape more salient than visual textural pattern (simply, color > shape > texture).

Language serves as the primary means for humans to describe visual features. Given the PSH, an interesting question arises: Whether the PSH can cast any influence on learning or processing the language structures used to regulate the relevant adjectives of those visual features. Answer to this question helps reveal the relationship between structural configuration in language and perceptual or cognitive constraints in humans, which is a challenging issue in modern psychology and linguistics (Christiansen and Kirby, 2003; Gentner and Goldin-Meadow, 2003; Hurford, 2007, 2012).

Many approaches have been adopted to study this issue. Corpus analyses have identified universal characteristics in language structures and potential links between language structures and cognitive constraints in humans (Ferrer-i-Cancho, 2004; Liu, 2010; Futrell et al., 2015). Computational modeling (Gong et al., 2013, 2014) has demonstrated how psychological or physiological constraints help shape word order (Gong et al., 2009), compositionality (Kirby, 1999; Brighton, 2002; Smith et al., 2003; Kirby et al., 2006), and syntactic patterns such as recursion, case, or long-distance dependency (Elman, 1990; Conway and Christiansen, 2001; Christiansen and Ellefson, 2002; Lupyan and Christiansen, 2002; Reali and Christiansen, 2009; Christiansen and Chater, 2015). In particular, some simulations show that the word order bias (in favor of certain orders like SOV or SVO but against others like VOS or OVS) in the world’s languages could result from individual perceptual constraint, which takes effect during communications (Gong et al., 2009). Other simulations illustrate that the universal color naming patterns in the world’s languages could result from the perceptual constraint of human eyes towards colors, which also takes effect during cultural transmission of color terms (Baronchelli et al., 2012). These studies have illustrated the effect of perceptual or cognitive constraints on structural configuration of language (Heine and Kuteva, 2008; Chater et al., 2009; Mesoudi, 2011; Richerson and Christiansen, 2013).

In experimental psychology, the paradigm of artificial language learning (ALL, in which participants are asked to learn a language or language-like system, and then tested on what they have learned; depending on underlying structures, ALL is also called artificial grammar learning) has been used to investigate issues concerning language and cognition (Esper, 1925; Reber, 1967; Folia et al., 2010; Onnis, 2012). An ALL experiment typically consists of a sequence of learning (a.k.a. training) and testing phases, which alternate throughout the experiment. In a learning phase, participants are presented with visual or auditory symbols concatenated following a predefined grammar-like structure. In the subsequent testing phase, they are presented with already-seen or unseen instances. Individual learning is said to occur when participants can distinguish instances that respect the underlying structure from those that violate it.

Artificial language learning experiments can design pseudo-words and structures distinct from participants’ native language to diminish the influence of participants’ prior linguistic knowledge and highlight corresponding learning mechanisms and factors hard to control in naturalistic scenarios (Onnis, 2012). They can also generate sufficient instances to trace individual learning and evaluate whether individuals can generalize their learned knowledge to unseen instances. In addition, by recruiting human participants and carefully designed artificial languages, ALL experiments can complement other approaches, such as verifying simulated behaviors and modeling results to bridge the gap between language processing in humans and relevant mechanisms in artificial agents (Kirby et al., 2008; Cornish, 2010). Furthermore, it has been repetitively shown that ALL experiments can uncover the same (or similar) mechanisms manifest in natural and artificial language processing (Reber, 1993; Gómez and Gerken, 2000; Pothos, 2007) and in first (Misyak et al., 2010; Misyak and Christiansen, 2012) and second language acquisition (Friederici et al., 2002; Robinson, 2010; Brooks et al., 2011; Petersson et al., 2012; Morgan-Short et al., 2014; Ettlinger et al., 2015). These advantages have made ALL experiments revitalize language learning research in the past century (Braine, 1963; Moeser and Bregman, 1972; Saffran et al., 1996; Morgan and Newport, 1981; Tily et al., 2011; Tabullo et al., 2012).

To our knowledge, there are no modeling or experimental studies that address directly the PSH and its influence on language learning. In this paper, we conducted an ALL experiment to study this issue. A number of artificial languages were designed, each describing two out of the three types of visual features in the PSH. In an artificial language, a visual feature was mapped to a phonetic segment, and segments, respectively, encoding the two features followed a consistent order. We referred to the theme-first principle in linguistics to clarify such orders. The principle states that more “thematic” information tends to precede less “thematic” one in normal linguistic expressions (Tomlin, 1986) (here, thematic information refers to the pragmatic or psycholinguistic reflex of the general attention in human cognition). This principle helps account for many cross-language phenomena, especially for word order (Halliday, 1967; Mathesius, 1975; Lambrecht, 1994; Cinque, 1999, 2010; Cinque and Rizzi, 2008; Longobardi and Guardiano, 2009). In terms of visual perception, it suggests that information of perceptually more salient feature should precede that of less salient feature. Following this principle, in our ALL experiment, we regarded an order as *congruent* with the PSH, if it puts a segment encoding a perceptually more salient feature in front of a segment encoding a less salient feature; otherwise, the order is deemed *incongruent*. We recruited human participants to learn, by repetitive exposure of instances, the artificial languages having congruent or incongruent orders, and assessed individual learning using seen and unseen instances.

In the following sections, we described the experiment, reported its results, and discussed the relation between language and human cognition based on this study.

## Materials and Methods

The experimental protocol was approved by the College Research Ethics Committee of University of Hong Kong. The methods were carried out in accordance with the approved guidelines from the College Research Ethics Committee. Informed consents were obtained from all participants.

### Participants

One hundred and thirty-two students from the University of Hong Kong participated into the experiment (66 females, mean age = 23.27, age range = 17–33, *SD* = 4.97). Forty Hong Kong dollars were paid to participants who had finished the experiment and filled in the post-experiment surveys. The recruited participants had normal or adjusted-to-normal vision, and reported no history of developmental delay or acquired neurological disorder. They were native Mandarin or Cantonese speakers, and had an intermediate level of English.

### Materials

**FIGURE 1:**
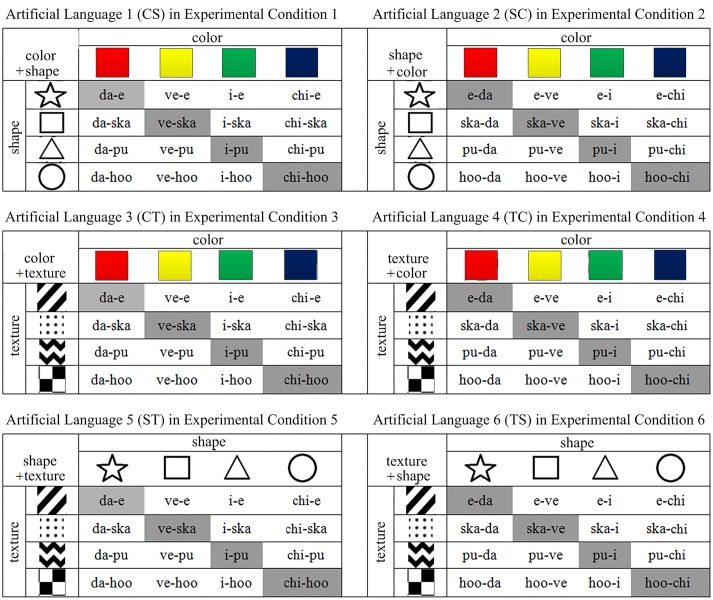
**Meaning-form mappings of the artificial languages in the six experimental conditions.** In each table, the rows and columns list the eight instances of the two types of visual feature (four in each type), and each cell shows the form encoding the stimuli having the features specified by the row and column. Each table shows the 16 meaning-form mappings of an artificial language. Hyphens in forms are added to highlight segments. In the actual experiment, participants are exposed to forms without hyphens or other indicators of structure.

We defined six artificial languages, respectively, used in six experimental conditions (see **Figure 1**). Each artificial language described two of the three visual features in the PSH (color (C) and shape (S), color and textural pattern (T), and shape and textural pattern). There are two reasons for considering orders between only two visual features. First, as shown in previous psychological experiments of visual feature saliency (Treisman, 1985; Callaghan, 1990; Healey and Enns, 1999; Healey, 2000), using languages encoding only two visual features can directly reflect whether the congruent or incongruent orders between the two affect the learning of those orders. Second, training participants on orders among three visual features would require more learning trials to give participants enough opportunities to detect and learn similarities between visual features and segments and similarities in regulating orders among segments. This would increase learning difficulty and memory burden, extend experiment time, and might have adverse effects on participants’ motivation.

Our training stimuli consisted of 48 images created by PhotoImpact X3. Each image depicted an object with a unique combination of a shape (star, square, triangle, or circle), a color (red, yellow, green, or blue), and a textural pattern (stripes, dots, zigzag, or checkerboard). These images were divided evenly into three sets. Each set of 16 images differed in two features (e.g., color and shape) and were the same in the third (e.g., texture) (see **Figure 1**).

All forms of the artificial languages were presented visually in the experiment. A form consisted of two compositional segments. A segment encoded one instance of a visual feature and had a consonant-vowel or vowel structure. All segments had roughly the same level of learning difficulty, and did not resemble any orthography of real words in English or any pronunciation of real characters in Mandarin or Cantonese. We also designed the segments to avoid iconicity (perceptuomotor analogies between aspects of a form and meaning of a word, e.g., onomatopoeia words and ideophones, Dingemanse, 2012; Dingemanse et al., 2015), which could assist language learning or comprehension (Simner et al., 2010; Pemiss and Vigliocco, 2014). In each form of an artificial language, the two segments followed a consistent order. Depending on encoded visual features, the order between segments was either congruent or incongruent with the PSH.

As shown in **Figure 1**, languages 1 and 2 describe color (C) and shape (S), languages 3 and 4 described color (C) and texture (T), and languages 5 and 6 described S and T. Each pair of the languages were formed by the same set of segments but differed in regulating order. Three of these languages (languages 1, 3, and 5) had congruent orders (CS, CT, and ST), and the other three (languages 2, 4, and 6) had incongruent orders (SC, TC, and TS).

### Procedure

The procedure was implemented using E-Prime 2.0. During the experiment, participants sat comfortably in front of a laptop in a bright, quiet room. They were asked to learn an “alien language” by viewing its meaning-form mappings displayed on a 21-inch computer monitor at a resolution of 1280 × 1024 and a refresh rate of 75 Hz. The font size was 64 pixels. The distance between the screen and participants’ eyes was approximately 64 cm. We used a between-subject design; each participant was assigned to one experimental condition to learn the corresponding artificial language. Gender and number of participants were balanced in each condition (11 females and 11 males). Prior to the experiment, the participants went through a two-minute familiarization block.

The experiment consisted of three 5-min blocks, with optional two-minute breaks in between; the whole experiment lasted about 20 min. A block consisted of a learning and a testing phase; in total, there were three learning and three testing phases to trace learning progress.

**FIGURE 2:**
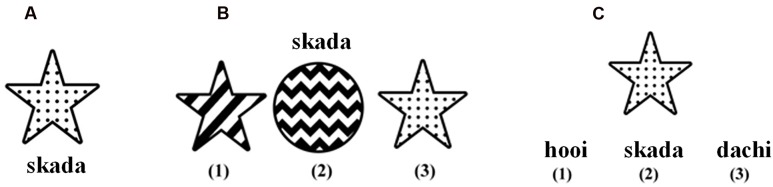
**Examples.** A training instance **(A)**, a meaning selection question **(B)**, and a form selection question **(C)**, which are taken from the experimental condition 6 (see **Figure 1**). The correct answer of the meaning selection question is image (3). The incorrect meanings share no or one (star shape) instance with the correct meaning. The correct answer of the utterance selection question is form (2). The incorrect forms share no or one segment (/da/) with the correct form, and form (3) uses an order (ST) distinct from the order (TS) of the artificial language.

In a learning phase, 12 out of the 16 meaning (image)-form mappings (those in the white cells in **Figure 1**) of an artificial language were displayed visually to participants. A mapping was shown on the center of the monitor, with the form presented simultaneously underneath the image (see **Figure 2A**). A mapping remained visible for five seconds. Presentation of all 12 mappings was repeated three times. Each time the meaning-form pairs were displayed in a pseudo-random order ensuring that the images of any two consecutively presented mappings shared no instances of the two types of visual features that the artificial language described. This setting prevented the participants from immediately noticing the associations between the visual features and the segments, thus increasing the difficulty of the learning task.

In a testing phase, individual learning was assessed by 20 forced-choice questions presented in a pseudo-random order. Participants gave their answers by key pressing. After the participants answered a question, the next one popped up without feedback. Ten of the questions were meaning selection questions (see **Figure 2B** for an example). In each of them, the participants saw a form followed by three meanings (images) displayed in a pseudo-random order. Participants were asked to select the image that they believed was expressed by the form. Incorrect meanings shared at most one instance of the visual feature with the correct one. The other ten questions were form selection questions (see **Figure 2C** for an example). In each of them, one image and three forms were displayed simultaneously. The participants were asked to select the form that they believed encoded the meaning. Incorrect forms shared at most one segment with the correct form. The segment orders in the incorrect forms were distinct from the order used in the instances in the learning phase.

The 12 meaning-form mappings shown in the learning phases appeared at least once and at most twice as the correct answers in the 20 testing questions. Each mapping had the same occurrence frequency in the learning and testing phases. To answer the testing questions correctly, the participants needed to learn not only the mappings between the visual features and the segments but also the order between the segments. Compared with the much larger search space in the free recall tasks as in previous studies (e.g., Cornish, 2010; Tamariz and Kirby, 2015), answers in the forced choice questions were more limited and allowed explicitly tracing the participants’ learning performances.

In the last testing phase, apart from the 20 normal testing questions containing the items already seen in the learning phases, there were additional four meaning selection and four form selection questions that contained the novel meaning-form mappings not presented in the learning phases (those in the gray cells in **Figure 1**). Performance on these items helped evaluate whether the participants could generalize their learned knowledge to unseen instances. All the 28 questions were presented in a pseudo-random order.

### Measures

In each testing phase, we recorded each participant’s accuracy (percentage of correct answers to the questions of the same type) and average reaction time to each of the meaning and form selection questions. In the last testing phase, apart from the measures to the normal testing questions, we also recorded the accuracies and average reaction times to the additional questions about the novel items. We grouped the accuracy and average reaction time data in the experimental conditions 1, 3, and 5 (the artificial languages therein had congruent orders) as the congruent set, and those in the conditions 2, 4, and 6 as the incongruent set. In each set, the accuracies and average reaction times were grouped according to the three testing phases. The measures to the additional questions formed the fourth phase. To meet the assumption of normality, we used the log-transformed (base *e*) reaction times in the analyses.

After the experiment, the participants were asked to fill a *post hoc* survey to indicate: which type of questions – meaning or form selection – was harder to answer; in which block they could confidently learn the “alien language”; and how difficult they felt to learn the “alien language” on a scale of 1 to 5, ‘1’ being the easiest, ‘3’ being neutral, and ‘5’ being the hardest.

### Preprocessing and Analyses

Following the general procedure in assessing experimental data (Osborne and Overbay, 2004), we removed the outliers from the accuracy and reaction time data before the analyses. Outliers were values exceeding 2.5 standard deviations from the group mean. For accuracies, outliers were accuracies that were too low; for reaction times, they were times either too long or too short. Among the 1056 (132 × 4 × 2) accuracy data in eight groups, 34 outliers were removed; for the reaction times, 23 were removed. Another way to handle outliers is to replace them with the group means, the results following this procedure were similar (see **Supplementary Table S1**).

We conducted two ANOVAs, respectively, on accuracy and average reaction time to test our working hypothesis that the PSH affects the learning of regulating orders between segments encoding the involved visual features. In the ANOVAs, we treated the congruency of artificial languages as a between-subjects factor (two levels: the congruent languages, those used in conditions 1, 3, and 5, and incongruent languages, those used in conditions 2, 4, and 6), and the experimental phase as a within-subjects factor (four levels: the testing phases 1, 2, 3, and 4, the latter of which consists of the measures to the additional testing questions involving the unseen items). The ANOVA tests also took into account the question type (two levels: meaning selection or utterance selection) and interaction between congruency and experimental phase. In addition to the ANOVA tests, we conducted group t-tests to compare the accuracies and average reaction times between the conditions differing in regulating orders (conditions 1 vs. 2, 3 vs. 4, and 5 vs. 6), which aimed to reveal possible learning biases for the congruent or incongruent orders. Following the Bonferroni correction, we set the critical *p* value to identify significant effects as 0.002 (0.05/(2+24), 26 tests in total). All the analyses were carried out in R 3.2.4 (R Core Team, 2016).

## Results

**Table 1** shows the results of the ANOVAs. In both tests, question type showed no significant effect, which matched the *post hoc* surveys; 125 participants felt invariant to both types of questions. This indicated that the way of recording the individual learning performance in our study had no obvious effect on the recorded results.

**Table 1:** Results of the ANOVAs of accuracy (ACC) and average reaction time (RT).

	ANOVA of ACC			ANOVA of RT		
		
Factor	*F*	*P*	η^2^	*F*	*p*	η*^2^*
Congruency	**30.413**	**<0.00005**	**0.029**	**17.442**	**<0.00005**	**0.017**
Phase	**40.006**	**<0.00001**	**0.106**	**24.090**	**<0.00001**	**0.066**
Question Type	0.819	0.366		0.558	0.455	
Congruency × Phase	4.439	0.004		2.867	0.036	


*Significant effects (whose *p*-values are below the critical *p*-value 0.002) are highlighted in bold.*

Both congruency and experimental phase showed significant main effects, but there was no significant interaction between the two. Compared with experimental phase, congruency had a smaller effect size η*^2^*. The significant effect of congruency confirmed our working hypothesis that the perceptual saliency hierarchy could affect individual learning of congruent or incongruent orders. The significant effect of experimental phase indicated that learning occurred at different experimental phases of instance exposure. The non-significant interaction between congruency and experimental phase suggested that the learning patterns across phases for the congruent and incongruent orders were largely the same.

**FIGURE 3:**
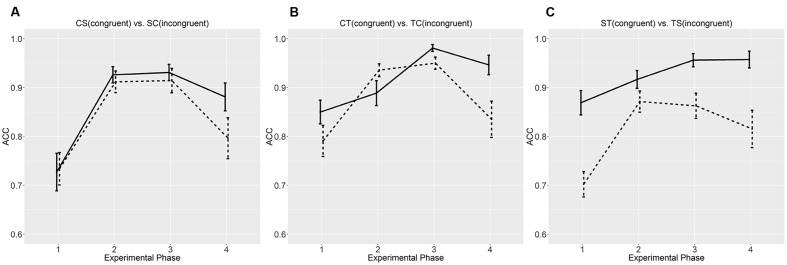
**Accuracies (ACC) at the four experimental phases.** **(A)** CS vs. SC; **(B)** CT vs. TC; **(C)** ST vs. TS. Error bars denote standard errors. Solid lines denote congruent orders (CS, CT, and ST), and dashed lines incongruent orders (SC, TC, and TS). “C”, “S”, and “T” stand for color, shape, and texture, respectively. “^∗^” marks significant difference based on group *t*-tests.

**FIGURE 4:**
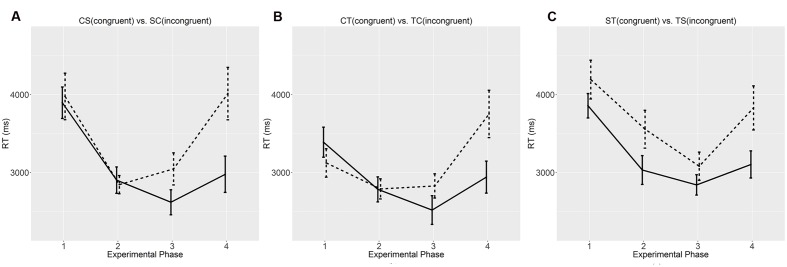
**Average reaction times (RT) at the four experimental phases.** **(A)** CS vs. SC; **(B)** CT vs. TC; **(C)** ST vs. TS. Error bars denote standard errors. Solid lines denote congruent orders (CS, CT, and ST), and dashed lines incongruent orders (SC, TC, and TS). “C”, “S”, and “T” stand for color, shape, and texture, respectively. “^∗^” marks significant difference based on group *t*-tests.

**Figures 3** and **4** compare the accuracies and average reaction times across the four phases between the conditions differing in regulating orders. Across the three phases, there were a general increase in accuracy and a general decrease in average reaction time, which echoed the significant effect of experimental phase in the ANOVAs. In our experiment, individual learning started at the first phase, and the improvement after the third phase was smaller than that after the second phase. After three phases, the participants had largely grasped the compositional languages in different conditions. These results also matched the *post hoc* surveys; 128 participants claimed that they had confidently learned most of the meaning-form mappings after the second phase, and the other four said that they had learned the language right after the first phase.

Although the participants claimed to have learned the artificial languages, their performances on the unseen items at the last phase revealed some biases for the congruent orders. As for the CS and SC orders, the participants showed similar accuracies, but their reaction times to the CS orders were shorter than those to the SC orders. As for the CT and TC orders, they showed higher accuracies and shorter reaction times to the CT order than the TC order. As for the ST and TS orders, they showed a similar bias for the ST order over the TS order. Significant difference in accuracy was also shown at the first and third testing phases concerning the seen instances. These biases also manifested in the *post hoc* surveys; when asked to evaluate the learning difficulty of the “alien language”, the average scores given by the participants in the CS and SC conditions were similar (2.38 vs. 2.64), but those in the CT and ST conditions were different (2.86 vs. 3.65), so were their scores in the ST and TS conditions (3.10 vs. 3.95).

## Discussion

In this paper, we evaluated whether the perceptual constraint regarding the saliency hierarchy of the basic visual features affects the learnability of ordering structures between the segments encoding such features in an artificial language. After repeated exposure to the tokens of the artificial languages with different orderings, the participants gradually learned the segments encoding color, shape, or textural patterns and the orders between these segments. Their judgements on the unseen instances indicated that they could generalize their learned knowledge and apply it to novel items. Moreover, they exhibited biases for the orders that were congruent with the perceptual saliency hierarchy regarding color, shape, and textural patterns. To be specific, they showed strong biases for the CT (color before textural pattern) and ST (shape before textural pattern) orders over the TC and TS orders, in terms of judging accuracy and average reaction time. Such biases started to exhibit during the learning process. They also showed a weak bias for the CS over the SC order, which only manifested in average reaction time when judging the unseen items.

In this ALL experiment, the observed biases were not induced by participants’ prior linguistic knowledge (of Mandarin, Cantonese, or English). In simple phrases of Mandarin or Cantonese, information of textural pattern often appears before that of shape, and color before shape (e.g., “*hongse* (red) *mutou* (wood) *yuan* (round) *zhuozi* (table)”) (Yip and Rimmington, 2004; Zhu, 2005), whereas the participants in our experiment exhibited a strong bias for the orders putting textural pattern after color or shape. In simple phrases of English, adjectives of shapes often appear in front of those of colors (e.g., “a round red wood table”) (Carter et al., 2011), but the participants showed no bias for color and shape at least in accuracy. In addition, the participants had no previous experience of the segments used in our experiment, and had no chance to apply their prior linguistic knowledge to change the artificial languages or develop one from scratch. These ensure that the observed patterns can be safely ascribed to the perceptual saliency hierarchy. Nonetheless, we acknowledge that participants’ alphabetic knowledge may potentially affect their performance in ALL. This is an inevitable limitation of ALL experiments recruiting alphabetic language speakers to learn alphabetic languages. Recruiting participants with no alphabetic experiences (e.g., pre-language children) or using uncommon symbols or non-linguistic forms to design artificial languages may help diminish such influence, as in experimental semiotics studies (Galantucci and Garrod, 2010, 2011) (e.g., Galantucci, 2005; Scott-Phillips et al., 2009; Taylor et al., 2011; De Boer and Verhoef, 2012; Claidière et al., 2014; Tamariz and Kirby, 2015). However, many of such studies focus on the emergence of a language-like communication system out of random signals, and participants therein are allowed to introduce signals that they prefer during the recall tasks.

Our fact that the perceptual saliency hierarchy affects the learning and processing of relevant language structures reveals a close relation between perceptual constraints in humans and structural configuration in language. In a linguistic form, if the ordering of segments encoding the visual features follows naturally the perceptual saliency of those features, production and comprehension of the form would be more straightforward. Then, compared with the forms having an incongruent order between those features, the accuracies of answering questions about the forms having congruent orders tend to be higher, and the reaction times shorter. This is evident by the strong biases for the CT and ST orders over TC and TS orders.

In addition, compared with color and shape, textural pattern is much less salient, and it is shown as contrast of color or shape. Detection of such pattern occurs after detection of color or shape, and relies on detection of color or shape (Treisman, 1985; Healey and Enns, 1999). This also explains the strong bias for the CT and ST orders. By contrast, color appears to be slightly more salient than shape, which resulted in the weak bias for the CS over SC order.

All these results are in line with the perspective that perceptual constraints affect the learning (and use) of related language structures (Heine, 2001; Pullum and Scholz, 2002; Mesoudi and Whiten, 2008; Christiansen and Chater, 2015). They also suggest that difference in saliency levels of the visual features could affect the degree of bias for the congruent orders regulating those features.

Given the bias for the congruent orders, a follow-up question arises: If the perceptual saliency hierarchy affects the regulating orders of the segments encoding the involved visual features, is this structure in all languages the same, favoring the congruent orders? The answer to this question is NO. Although there lack large-scale typological studies of adjective orders in world’s languages, as shown in simple Chinese expressions, the adjectives of textural patterns usually appear in front of the color or shape adjectives. Although in many English phases, the shape adjectives should appear in front of the color adjectives, most people have a relatively free order between the two. Considering these, apart from the perceptual saliency hierarchy, the structural configuration of language is also subject to other constraints. One candidate of such constraints comes from the socio-cultural environment of language. As shown in typological studies of structural diversity in world’s languages (Haspelmath, 2007; Evans and Levinson, 2009; Dunn et al., 2011), cultural histories of speakers and contact histories between different languages could induce different types of structure.

In addition, our experiment showed that despite the fact that the participants exhibited biases towards the congruent orders, after a small number of learning rounds, they could largely grasp both the biased and unbiased orders, reaching high (over 0.8) accuracy and short reaction time. Following the dynamics in the three experimental phases, we can reasonably expect that given more rounds of learning the participants would learn each artificial language equally well, no matter whether its order was congruent or incongruent. This suggests that the structures distinct from the biased ones can be equally acquired by speakers. This makes sure that other types of structures, once induced due to other constraints, can also be transmitted across generations of leaners.

The above discussion reveals a complicated relation between language and perception.

On the one hand, during cultural transmission of language across multiple generations of learners, individuals’ perceptual constraints could favor some structures congruent with the perceptual constraints, thus causing a bias towards those structures. This has been demonstrated in many experimental and simulation studies. For example, some experiments have shown that the dominant word orders in world’s languages are also easier to learn (Culbertson et al., 2012; Fedzechkina et al., 2012; Culbertson and Adger, 2014). Some simulations have also revealed that cultural transmission could amplify small biases for certain structure and make it prevalent in communal languages of later generations (Griffiths and Kalish, 2007; Kirby et al., 2008; Smith, 2011).

On the other hand, other factors, such as different socio-cultural histories could induce distinct language structures. Some modeling studies have demonstrated that socio-cultural interactions could trigger a variety of structural forms, which can be equally acquired and transmitted by generations of language learners (Steels and Belpaeme, 2005; Steels, 2011, 2012). More importantly, as shown in our study, if different structures are more or less functionally equivalent, they can be acquired equally well by speakers, given sufficient rounds of learning. This may diminish the bias for certain structures to a certain extent, and lead to diversity in structural configuration of language.

These two aspects suggest that the actual structures in different languages have arisen as a compromise between both the individual perceptual constraints and the socio-cultural factors (Christiansen and Chater, 2008; Liu, 2014). Such compromise leads to a biased distribution of languages predominantly in certain structures. The mutual influence of individual and socio-cultural factors has been illustrated in some simulation studies of word order bias (Gong et al., 2009) and color naming patterns (Baronchelli et al., 2012).

Our experiment, as an individual learning experiment, could not fully demonstrate the mutual influence of individual and socio-cultural aspects. Nonetheless, it confirmed the influence of individual perceptual constraint (i.e., the perceptual saliency hierarchy) on learning congruent and incongruent orders. It also revealed that given more trials the learning of incongruent orders could reach a similar level to the learning of congruent orders. This suggested that the structures induced by other factors, even though conflicting to the structures favored by perceptual constraints, could still be acquired and transmitted. Both of these findings can shed light on the relation between individual learning and cultural transmission, and contribute to the discussion of the causal factors for the structural diversity in languages (Longobardi and Guardiano, 2009).

Finally, some aspects of this study can be extended in future work. For example, we may recruit pre-language or language-learning children to further diminish the influence of individuals’ prior linguistic knowledge (Saffran et al., 2007; Folia et al., 2010). Compared with visual presentation, auditory presentation better resembles language exchange in everyday life. It may reduce the effect of orthography in language learning, though not eliminating it altogether (Cuskley et al., 2015). In auditory presentation, factors such as memory (Morrison et al., 2014), stress or prosody may also modulate the biases for certain structures.

## Ethics Statement

The experimental protocol was approved by the College Research Ethics Committee of University of Hong Kong. The methods were carried out in accordance with the approved guidelines from the College Research Ethics Committee. Written informed consents were obtained from all participants.

## Author Contributions

TG and LS designed the research, YL carried out the study. TG and YL analyzed the results. TG, LS, and YL wrote the paper.

## Conflict of Interest Statement

The authors declare that the research was conducted in the absence of any commercial or financial relationships that could be construed as a potential conflict of interest.
