# Supplementary material for: Influence of Perceptual Saliency Hierarchy on Learning of Language Structures: An Artificial Language Learning Experiment
Source: Front Psychol. 2016 Dec 21;7:1952. doi: 10.3389/fpsyg.2016.01952 (PMC5174136; doi:10.3389/fpsyg.2016.01952)
Supplement: Supplementary file 1 [file Table_1.docx]

**Table S1. Results of the ANOVAs of accuracy (ACC) and average reaction time (RT) based on the dataset using group means to replace outliers.**

|  | ANOVA of ACC | | | ANOVA of RT | | |
| --- | --- | --- | --- | --- | --- | --- |
| **Factor** | ***F*** | ***P*** | ***η^2^*** | ***F*** | ***p*** | ***η^2^*** |
| Congruency | **31.698** | **< .00005** | **.026** | **17.495** | **< .00005** | **.015** |
| Phase | **41.490** | **< .00001** | **.102** | **25.225** | **< .00001** | **.065** |
| Question Type | .858 | .355 |  | .558 | .455 |  |
| Congruency × Phase | 4.632 | .003 |  | 3.073 | .027 |  |

*Significant effects (whose p values are below the critical p value .002) are highlighted in bold.*
